# Biomechanical markers associations with pain, symptoms, and disability compared to radiographic severity in knee osteoarthritis patients: a secondary analysis from a cluster randomized controlled trial

**DOI:** 10.1186/s12891-022-05845-1

**Published:** 2022-10-05

**Authors:** Fatima Bensalma, Nicola Hagemeister, Alix Cagnin, Youssef Ouakrim, Nathalie J. Bureau, Manon Choinière, Neila Mezghani

**Affiliations:** 1 Université TÉLUQ, Centre de Recherche LICEF, 5800 Saint-Denis Str., #1105, Montreal, QC H2S 3L4 Canada; 2 École de Technologie Supérieure, Laboratoire de Recherche en Imagerie Et Orthopédie, 900 Saint-Denis Str., R11-430, Montreal, QC H2X 0A9 Canada; 3 Centre Hospitalier de L’Université de Montréal, 900 rue Saint-Denis, Montreal, QC H2X 0A9 Canada

**Keywords:** Biomechanical markers, Knee osteoarthritis, Pain, Disability, X-Ray grading, Canonical correlation analysis

## Abstract

**Background:**

Conventional radiography is commonly used to diagnose knee osteoarthritis (OA), but also to guide clinical decision-making, despite a well-established discordance between radiographic severity and patient symptoms. The incidence and progression of OA is driven, in part, by biomechanical markers. Therefore, these dynamic markers may be a good metric of functional status and actionable targets for clinicians when developing conservative treatment plans. The aim of this study was to assess the associations between biomechanical markers and self-reported knee function compared to radiographic severity.

**Methods:**

This was a secondary analysis of data from a randomized controlled trial (RCT) conducted in primary care clinics with knee OA participants. Correlation coefficients (canonical (ρ) and structural (*Corr*)) were assessed between the Knee Injury and Osteoarthritis Outcome Score (KOOS) and both, radiographic OA severity using the Kellgren-Lawrence grade, and three-dimensional biomechanical markers quantified by a knee kinesiography exam. Significant differences between coefficients were assessed using Fischer’s z-transformation method to compare correlations from dependent samples.

**Results:**

KOOS and biomechanical data were significantly more associated than KOOS and X-ray grading (ρ: 0.41 vs 0.20; *p* < 0.001). Structural correlation (*Corr)* between KOOS and X-ray grade was 0.202 (4% of variance explained), while individual biomechanical markers, such as the flexion during loading, explained up to 14% of KOOS variance (i.e., *Corr*^*2*^). Biomechanical markers showed the strongest associations with Pain and Activity of Daily Living KOOS subscales (both > 36% variance explained), while X-ray grading was most associated with Symptoms subscale (21% explained; all *p* ≤ 0.001).

**Conclusions:**

Knee biomechanical markers are associated with patient-reported knee function to a greater extent than X-ray grading, but both provide complementary information in the assessment of OA patients. Understanding how dynamic markers relate to function compared to radiographic severity is a valuable step towards precision medicine, allowing clinicians to refine and tailor therapeutic measures by prioritizing and targeting modifiable biomechanical markers linked to pain and function.

**Trial registration:**

Original RCT was approved by the Research Ethics Boards of École de technologie supérieure (H20150505) and Centre hospitalier de l’Université de Montréal (CHUM-CE.14.339), first registered at https://www.isrctn.com/ (ID-ISRCTN16152290) on May 27, 2015.

## Background

Knee osteoarthritis (OA) is a leading cause of disability among older adults and is characterized by morphological changes in joint structure, joint pain, mechanical joint dysfunction, and muscle weakness [1]. Due to the multi-factorial nature of this disease, associations between patient clinical characteristics and objective measures of disease are complex. Typically, radiographs are used to complement the findings from a clinical evaluation of patients with suspected knee OA [2]. The radiographic assessment is largely used for diagnostic purposes, but these images are also used to guide clinical decision-making [3, 4]. Although radiography is a suitable tool to assess static joint structure, there are important limitations when using these results to guide and develop conservative treatment plans.

Patient symptoms are often not correlated with radiographic severity [5, 6]. Patients with severe pain can present minimal radiographic severity, and conversely, those with severe radiographic OA may present with minimal symptoms [7]. This may partially explain why clinicians have identified problems in the conventional approach to determining optimal treatment strategies and reported the need of additional tools to help them choose the most effective treatment [3, 8]. Although guidelines and reviews recommend using radiographs as part of a multi-factorial examination, radiographs often constitute the primary method upon which treatment plans are made, despite providing minimal leads to assist clinicians in the development of treatment plans [9, 10]. Pain, severity of disability, and other patient-specific factors should also be considered to address this clinical gap in precision medicine for this patient population and prevent that X-ray supplant other clinical features in decision-making [10–14].

In addition to joint pain and disability, most patients with knee OA demonstrate altered knee biomechanics during gait [15]. These changes are typified by reduced excursion in the sagittal plane (i.e., flexion–extension), dynamic misalignments in the frontal plane (i.e., adduction/abduction [clinically known as varus/valgus]), as well as altered rotations in the transverse plane (i.e., internal/external rotation) [16–19]. Biomechanical alterations are a central aspect of knee OA; they not only serve as biomechanical markers of OA severity but are also predictive of its onset and future progression [20, 21]. As the disease progresses, there is a decrease in joint excursions and an increase in dynamic frontal plane misalignments [16, 22, 23]. Patients with a limited dynamic knee range of motion, greater medial compartment loading, and varus thrust (i.e., sudden lateral shift of the knee during the loading phase of the gait) in the frontal plane at baseline are at greater risk for symptomatic and radiographic progression in the future [18, 24–27]. Interventions that specifically target altered biomechanics can reduce symptoms of knee OA, have the potential to delay the need for knee surgery, and alter the risk of structural OA progression [28–31]. It is important to note that these biomechanical markers can be modified through conservative treatment plans, making them ideal actionable targets for effective treatment [32, 33].

Given the associations between biomechanical markers and OA progression, evaluating and treating the biomechanical dysfunction of patients with knee pain is a medical necessity for optimal management [34]. Knee radiographic measures are limited predictors of dynamic joint alignment during weight-bearing activities. Indeed, an accurate, objective, three-dimensional assessment of the patient’s lower limb biomechanics is essential to fully understand her/his gait profile and the risk of OA progression [35–37].

Considering the importance of biomechanical markers as risk factors for OA progression and their modifiable nature, they may be a good metric of functional status and actionable targets for treatment plans that may be used to complement the standard radiographic exam as objective data. However, the relationship between symptoms, pain, disability, and biomechanical characteristics is not well understood. If biomechanical markers were to be considered meaningful measures to support and guide conservative treatment plans, these metrics 1) must be significantly associated with self-reported knee function and 2) provide information that is distinct from a standard radiographic exam. Therefore, the aim of this study was to assess the associations between biomechanical markers and self-reported knee function compared to radiographic severity.

## Methods

### Subjects

This study was a secondary analysis of baseline data from a randomized controlled trial (RCT; ISRCTN16152290) evaluating the clinical utility of a knee kinesiography assessment as part of knee OA patients’ care pathway. Details on participant recruitment and enrollment are documented elsewhere [31]. Briefly, patients seeking care for knee pain were recruited in primary care clinics in the Province of Quebec (Canada). Patients were eligible if they were diagnosed with knee OA after a clinical assessment and if they 1) reported knee pain ≥ 4/10 on a numeric pain scale in the past seven days, 2) had Kellgren-Lawrence (KL) radiographic severity scores ≥ 2, and 3) were not on a waiting list for a total knee arthroplasty (TKA) [38]. The exclusion criteria were inflammatory arthritis, active cancer, or pregnancy. When both knees were diagnosed with OA, only data from the most painful knee was used for this analysis. All subjects signed informed consent. The study protocol was approved by the Research Ethics Boards of the École de technologie supérieure (H20150505) and the Centre hospitalier de l’Université de Montréal (CE.14.339) prior to participating in the RCT.

### Radiographic assessment

Kellgren-Lawrence (KL) X-ray grade was determined using conventional radiographs by an experienced fellowship-trained musculoskeletal radiologist. Grades were based on several radiographs including 1) a weight-bearing anteroposterior view of both knees, 2) a non-weight-bearing lateral view, and 3) an axial view of the affected knee. The KL score is a reliable method to grade OA severity and has been used in numerous clinical studies [38–40].

### Knee kinesiography examination

**Fig. 1:**
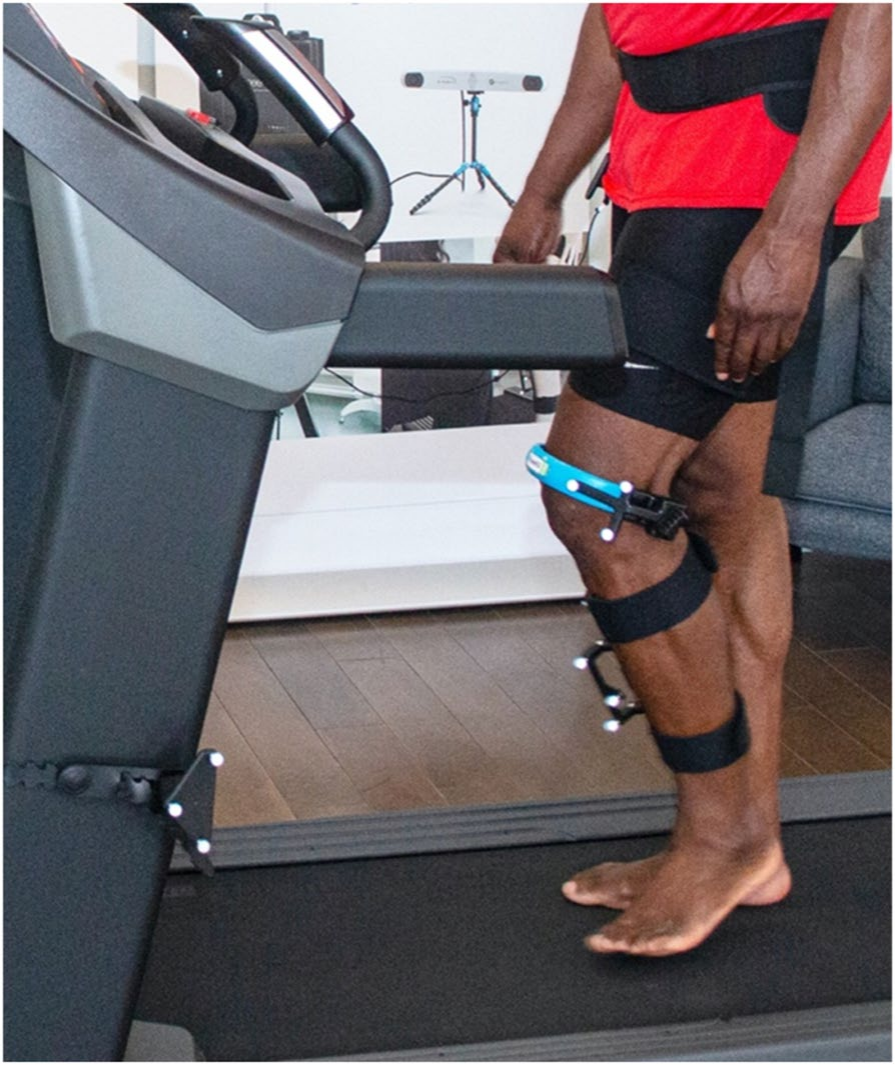
Patient wearing the KneeKG® exoskeleton tracking device during the knee kinesiography exam

Knee biomechanical markers were measured during a knee kinesiography examination with the KneeKG® system (Emovi Inc.). This is a portable, validated, FDA (510 k) cleared, Health Canada licensed and CE marked system, appropriate to assess three-dimensional (3D) knee kinematics in the clinical setting [41]. The global 3D positions of retroreflective markers affixed to the distal thigh, lower leg, and waist of an exoskeleton are tracked through an infrared camera (Fig. 1).

**Fig. 2:**
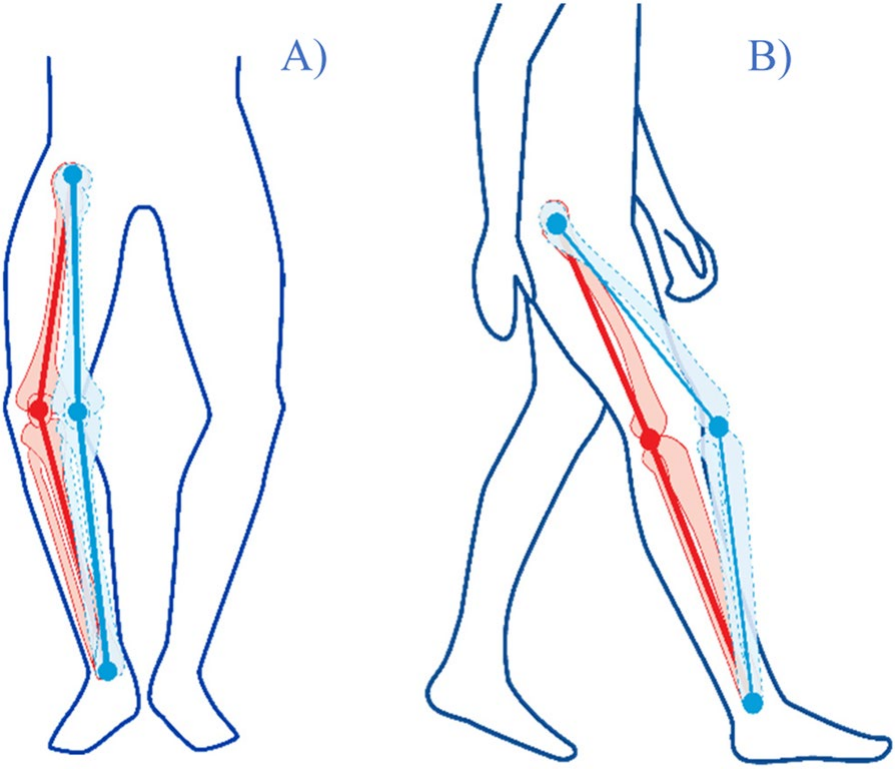
Illustrations of two dynamic biomechanical markers associated with OA progression. **A** Dynamic varus alignment (in red) is defined as a lateral offset of the knee during gait. A healthy knee is expected to maintain a more neutral position during motion (blue line). **B** Reduced knee flexion excursion during loading (in red) is characterized by limited flexion of the knee during loading. This occurs during the phase in which the knee acts as a shock-absorber. A healthy knee is expected to have substantial flexion (dashed blue line) to adequately absorb the loads induced by the body weight

It identifies and analyzes biomechanical markers in all three planes of movement (i.e., flexion/extension, varus/valgus, and internal/external rotation). After a short familiarization period, subjects walk for two bouts of 45 s on a treadmill at a self-selected comfortable walking speed. From the 15 most repeatable gait cycles (i.e., between two heel strikes on the ground of the same leg), a mean kinematic curve was calculated for each plane of movement. In this study, seventy biomechanical parameters were extracted from the 3D knee kinematic curves. These variables included common measures such as peak joint angles, joint excursions, and joint positions at relevant discrete time points and intervals (e.g., at initial contact, during loading, at push-off, etc.; see examples in Fig. 2).

### Self-reported knee function

All subjects completed the Knee Injury and Osteoarthritis Outcome Score (KOOS) patient-reported outcome measure. This is a valid and reliable instrument to evaluate knee OA patients, which has been validated for the French-speaking population [42–44]. The KOOS evaluates knee OA impact over five domains, or subscales, including Symptoms, Pain, Activities of daily living (ADL), Sports and recreation (SPORT), and Quality of life (QOL). Scores on the subscales range from 0 (extreme symptoms) to 100 (no symptoms).

### Statistical analysis

Descriptive statistics for patient characteristics were calculated. The relationships between X-ray grade, biomechanical markers, and KOOS score were evaluated using a canonical correlation analysis (CCA). This type of analysis is a statistical multivariate method analogous to a regression analysis to determine the association between two sets of variables derived from the same group of individuals instead of between two variables [45]. The associative results of this approach are an extension of a bivariate approach in which Pearson’s correlation coefficient (*r)* represents the strength of the relationship between two variables. In CCA, Pearson's coefficient is maximized between two sets of variables, allowing the quantification of two correlations. The first is the canonical correlation (i.e., ρ coefficient) that quantifies the global association between the two sets. The ρ coefficient ranges from 0 to 1 because of the standardized weights used to build the sets of variables. The second is the structural correlation (i.e., *Corr* coefficient), which estimates the association between a set as a whole and each variable of the other set. The *Corr* coefficients range from -1 to 1 and are simply Pearson *r* statistics, thus indicating the direction of the association. The squared values of these two coefficients are analogous to any other *r*^*2*^-type effect size and indicate the proportion of variance shared between the two sets or between a variable of one set and the other set as a whole (i.e., ρ^2^ and *Corr*^2^ respectively) [46].

Two canonical correlation models were run, one between the KOOS set (i.e., the 5 sub-scores) and the X-ray grade set (i.e., the 3 grades), and another one between the KOOS set and the biomechanical set (i.e., the 64 non-redundant biomechanical parameters out of 70). The significance of each canonical model was assessed using Pillai’s trace. Significant differences between coefficients were assessed using a method based on Fischer’s z transformation to compare correlations from dependent samples [47, 48]. P-values less than or equal to 0.05 were considered significant. All analyses were performed with the R software (version 3.6.3.) [49].

## Results

Complete data from 415 participants (251 women and 164 men) were available for this analysis. The mean (± standard deviation) age and body mass index were 63.3 ± 9.2 years and 30.3 ± 5.6 kg/m^2^, respectively. Half of the participants had bilateral OA (*N* = 211/415: 50.8%). The radiographic severity was evenly distributed among patients in the sample (KL 2 = 137, KL 3 = 149, and KL 4 = 129). Mean KOOS scores for men and women are shown in Table 1.

**Table 1:** Mean (± standard deviation) for KOOS subscales in men, women, and by KL grade

	**Symptoms**	**Pain**	**ADL**	**SPORT**	**QOL**
All subjects	62.41 ± 1.66	58.99 ± 1.70	65.48 ± 1.87	35.88 ± 2.46	49.49 ± 2.28
*KL 2*	65.65 ± 1.65	61.48 ± 1.88	67.47 ± 1.97	40.95 ± 2.58	52.01 ± 2.39
*KL 3*	62.37 ± 1.69	59.12 ± 1.69	65.41 ± 1.93	35.70 ± 2.49	49.20 ± 2.35
*KL 4*	59.00 ± 1.59	56.21 ± 1.47	63.44 ± 1.66	30.70 ± 2.18	47.14 ± 2.07
Women	61.70 ± 1.63	58.11 ± 1.69	64.60 ± 1.92	35.74 ± 2.50	50.17 ± 2.29
Men	63.48 ± 1.72	60.35 ± 1.71	66.83 ± 1.78	36.10 ± 2.39	48.44 ± 2.27

*KOOS*: 0 = extreme symptoms, 100 = no symptoms, *ADL* Activities of daily living, *SPORT* Sports and recreation, *QOL* Quality of life

### Canonical correlations

The two canonical correlation models were run to include all patients, and for women and men separately. Table 2 displays the ρ coefficient and Pillai’s trace *p*-values associated with each model. As significant relationships were found between KOOS scores and X-ray grade, as well as between KOOS scores and biomechanical parameters, Fischer’s z scores and associated p-values were calculated to compare ρ coefficients. The association between KOOS and biomechanical sets was significantly stronger than the association between KOOS and X-ray grade sets, and this was true both in men and women (all *p* < 0.001; Table 2). X-ray grades only explained between 2 and 5% of the variance in KOOS scores, while biomechanical parameters explained between 17 and 30% of this variance (i.e., ρ^2^ values). Given these results, the following structural *Corr* coefficient analysis was conducted to include men and women.

**Table 2:** Comparisons of canonical ρ coefficients between the KOOS and the two data sets

	KOOS & X-ray grade (*ρ* and Pillai’s trace p-value)	KOOS & Biomechanical parameters (*ρ* and Pillai’s trace p-value)	Comparison using Fischer’s z (p and z values)
All subjects *(N* = *415)*	*ρ* = 0.20 (*p* = 0.005)	*ρ* = 0.41 (p < 0.001)	*p* < 0.001* (z = -4.393)
Women *(N* = *251)*	*ρ* = 0.14 (*p* = 0.016)	*ρ* = 0.50 (p < 0.001)	*p* < 0.001* (z = -6.652)
Men *(N* = *164)*	*ρ* = 0.23 (*p* = 0.030)	*ρ* = 0.55 (p < 0.001)	*p* < 0.001* (z = -5.609)

### Structural correlations

**Fig. 3:**
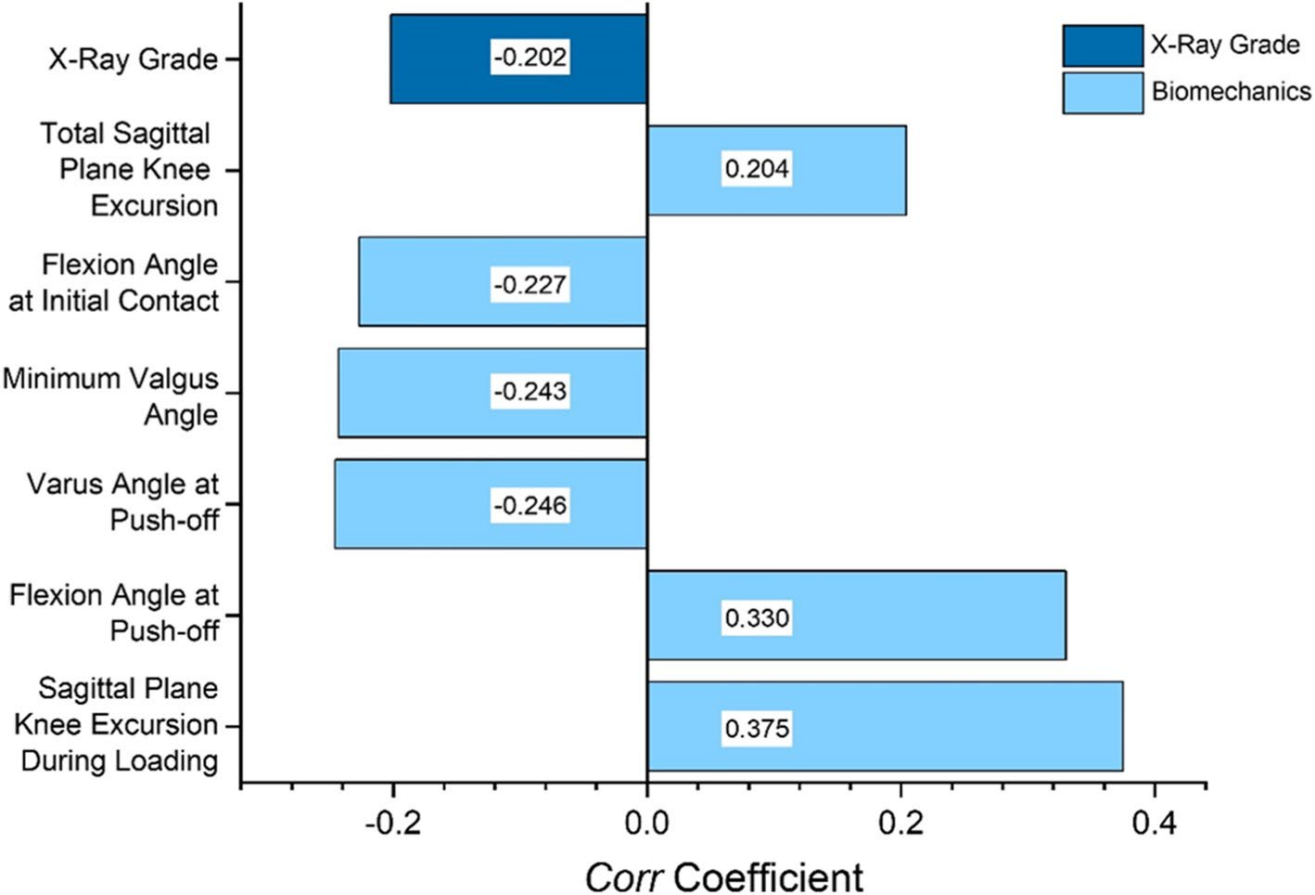
*Corr* coefficients between X-ray grades and individual biomechanical parameters and the KOOS set

Figure 3 shows the structural *Corr* coefficients between each variable from X-ray and biomechanical sets and the KOOS set as a whole. For the sake of clarity, only *Corr* coefficients above |0.2| are displayed (the higher the KOOS, the better the knee function).

The *Corr* coefficient was -0.202 for the KOOS and X-ray grades. It ranged from 0.204 (total sagittal plane knee excursion) to 0.375 (flexion excursion during loading) for the KOOS and the six individual biomechanical markers with the strongest association. Not surprisingly, a higher X-ray grade was associated with lower (i.e., worse) KOOS scores. Greater knee flexion angle at initial contact and knee varus angle during gait, especially at push-off, were also associated with lower KOOS scores. Greater sagittal plane knee motion, especially during loading, and greater knee flexion angle at push-off were associated with higher (i.e., better) KOOS scores (see Fig. 3).

**Fig. 4:**
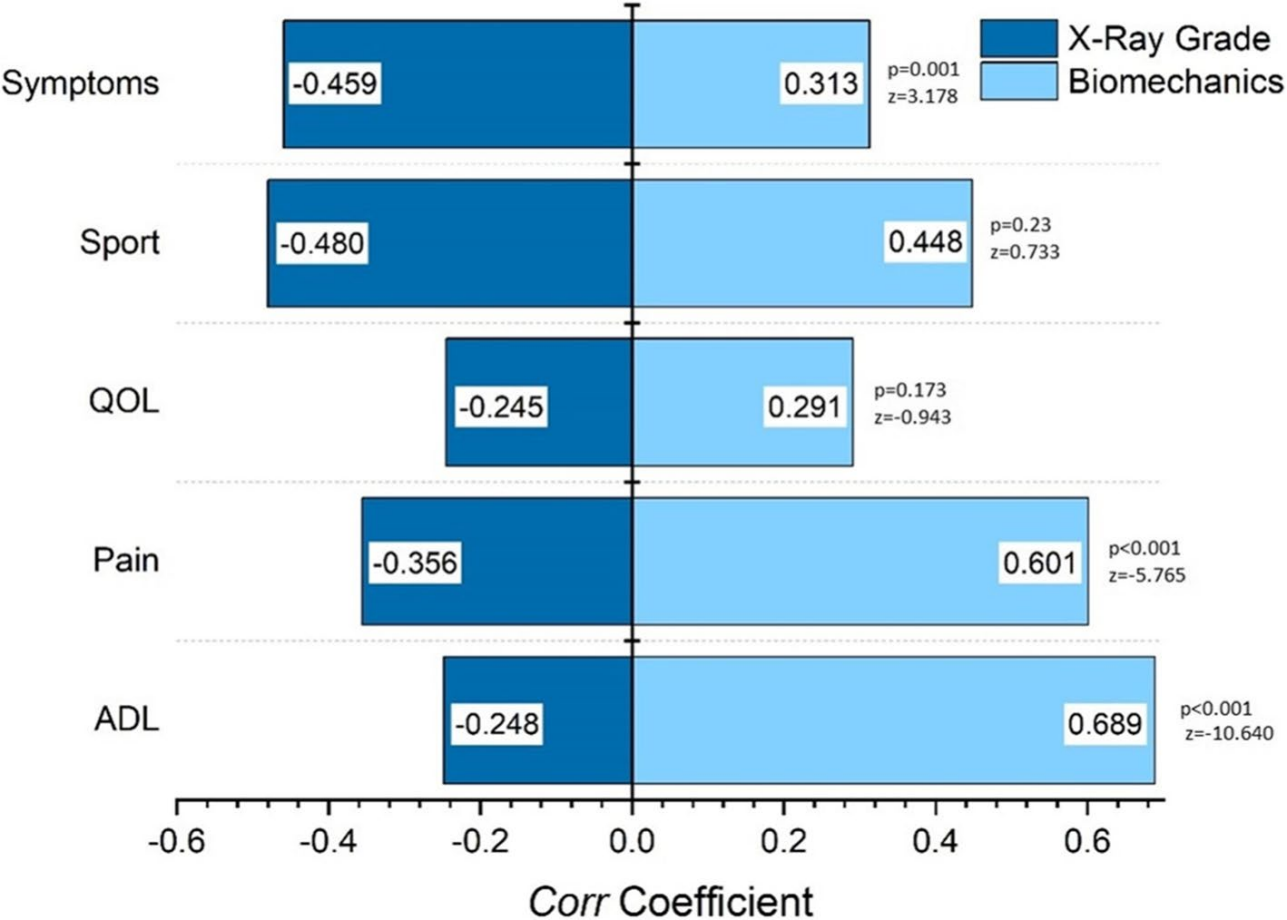
*Corr* coefficients between each KOOS subscale and X-ray grade and biomechanical sets

Structural *Corr* coefficients between each KOOS sub-score and the two other sets are displayed in Fig. 4. The strength of these associations differed depending on the KOOS subscale. The strongest associations for biomechanical markers occurred with the Pain and ADL KOOS subscales (both *Corr* > 0.6; more than 36% of variance explained), while the strongest association for X-ray grade occurred with the Symptoms and SPORT KOOS subscales (both *Corr* > 0.45; more than 20% of variance explained). However, *Corr* coefficients were only statistically different between X-ray grade and biomechanical sets on the Pain, ADL, and Symptoms subscales (all *p* ≤ 0.001 with Fischer’s z scores; Fig. 4).

## Discussion

Biomechanical markers, taken altogether, are more strongly associated with self-reported knee function scores than radiographic severity in OA patients. Considering that they provide distinct information from standard radiographs, as the associations between biomechanical markers and knee function were significantly different compared to radiographic severity, as well as their importance in the onset and progression of the disease and their modifiable nature, these results strengthen previous findings that support the use of biomechanical markers to support and guide the development of targeted conservative treatment plans.

Their superior relationship with self-reported knee function was most obvious when the KOOS was evaluated as a whole (e.g., collapsed subscales; Table 2) as the association between KOOS and biomechanics was significantly stronger than the association with X-ray grading. At the structural level, six individual biomechanical markers from the sagittal and frontal planes were more associated with the KOOS than the X-ray grade (Fig. 3). Furthermore, biomechanical markers had significantly stronger associations with Pain and ADL scores, whereas X-ray grades were more strongly associated with Symptoms scores (Fig. 4). Taken altogether, these results suggest that biomechanics and imaging could play a complementary role in describing the impact of OA, as the X-ray grade may be more associated with symptoms. Nonetheless, the proportion of variance explained by these associations remains moderate, underscoring that neither biomechanics nor imaging should be used independently to accurately assess an individual’s function.

The association between biomechanical markers and KOOS was notable, explaining up to 30% of variance. In knee OA patients, there is a clear relationship between movement patterns and functional ability [50, 51]. Patients with more normal gait patterns have greater mobility and better scores on performance-based functional assessments [52]. This may be the driving factor as to why biomechanical markers have a greater association with ADL subscales, compared to X-ray grading. These findings also support the notion that radiographic severity may not be the most accurate assessment of the patient’s level of joint pain. Meanwhile, the reported association between biomechanics and pain has also been reported in previous studies [53–55]. Thus, integrating a dynamic assessment of gait patterns as part of clinical examination may help design treatment plans that target pain and function.

The fact that the association with X-ray grading was maximized for the Symptoms subscale may be attributed to the questions in this subscale, which focus on knee joint locking, crepitus, swelling, and stiffness. These are all attributes that may be associated with structural changes within the joint and may explain why the association with X-ray grading, a measure of structural change, was higher for this subscale. This echoes a 2010 study from Fukui et al. where a significant association was reported between radiographic joint space width and medial joint line tenderness [56]. Interestingly, the lowest association for both biomechanics and X-rays was with the QOL subscale. It is possible that other psychological, societal, or economic factors influenced the scores on this subscale. It also only includes four questions and thus may not fully capture the symptomatic, structural, or biomechanical drivers of quality of life.

The strongest association between individual biomechanical markers and KOOS was found for the flexion excursion during loading, as greater excursion was associated with better KOOS scores (*Corr* = 0.375; Fig. 3). Previous studies already reported significant correlations between this biomechanical marker and OA severity and overall health status [20, 57]. Furthermore, it has been identified as a predictor of future TKA and identified as a residual deficit that does not resolve after TKA [13, 58]. Thus, patients with limited range of motion in flexion during loading are more likely to have more advanced structural OA, as well as being at greater risk for future knee replacement. Dynamic frontal plane position was also significantly associated with the KOOS (Fig. 3), as greater varus angles, especially at push-off, were associated with worse KOOS scores. Clinically, this suggests that a more neutral alignment during dynamic walking is associated with better outcomes. Added to the importance of dynamic knee alignment as a predictor of OA progression, these results support the relevance of focusing on the identification and correction of sagittal and frontal plane knee dysfunctions during weight-bearing activities as part of conservative management for patients with knee OA [59]. A dynamic assessment of biomechanical markers could thus be added to the clinical evaluation. as recommended by clinical guidelines [60], in complement to both X-ray and self-reported data (e.g., KOOS). Based on patients’ established profile and physician’s objectives, targeted therapies (i.e., movement retraining, personalized therapeutic exercises, bracing etc.) could aim at addressing identified biomechanical markers through a personalized treatment plan. Considering the added value of their associations with pain and function compared to X-ray, biomechanical markers with the highest *Corr* coefficients (Fig. 3) could be address in priority to impact specific patient-reported outcomes. Clinical implications of the use of biomechanical markers in conservative care are detailed in the original RCT [31].

There are some limitations to this work that should be addressed. First, this was a secondary analysis from an RCT and the inclusion and exclusion criteria narrowed the window of eligible participants. Patients with early-stage OA with little or no pain and those with late-stage OA on the waiting list for TKA were not included in the study sample. Therefore, these results may only be generalizable to patients with at least moderate pain (≥ 4/10) and not to patients with very mild symptoms or presenting with low radiographic severity (KL < 2). Second, results were not stratified by gender, age, OA bilaterality or affected knee compartment (i.e., medial/lateral), which could have allowed to better understand how the associations may evolve between different patient’s phenotypes. It is also important to remember that the aim of canonical correlation analyses is to describe and determine the linear relationships between two sets of variables without establishing a causality. The values ​​of canonical and structural correlations are calculated in multivariate cases which cannot be directly compared with those obtained in bivariate cases. In fact, CCA seeks the maximum correlation with a linear combination of the variables, as opposed to multiple regression where it is only a single dependent variable. Therefore, these absolute values ​​do not usually come close to a value of 1, as seen in bivariate correlations. Although the squared values of ρ and *Corr* coefficients are analogous to multiple regression’s R^2^, there are no generally accepted guidelines on the practical significance of these statistical measures in the context of canonical relationships [61]. Thus, the proportion of explained variance should be interpreted carefully.

## Conclusions

Knee biomechanical markers are associated with patient-reported knee function to a greater extent than X-ray grading. More specifically, biomechanical markers were more associated with pain and function during activities of daily living, while imaging was more associated with symptoms. Results support the use of a complementary objective biomechanical evaluation during clinical assessment to obtain a more complete functional profile of knee OA patients. This complementary information may constitute a valuable step towards precision medicine, allowing clinicians to rely on modifiable targets to tailor therapeutic measures to the specificities of each patient.

## Data Availability

The datasets generated and/or analyzed during the current study are not publicly available due to ethics committees’ restrictions but are available from the corresponding author on reasonable request.

## Abbreviations

OA: Osteoarthritis
RCT: Randomized controlled trial
KL: Kellgren-lawrence
TKA: Total knee arthroplasty
3D: Three-dimensional
KOOS: Knee injury and osteoarthritis outcome score
ADL: Activities of daily living
SPORT: Sports and recreation
QOL: Quality of life
CCA: Canonical correlation analysis

## References

[1] Zhang Y, Jordan JM (2010). Epidemiology of osteoarthritis. Clin Geriatr Med.

[2] Claessens AAMC, Schouten JSAG, Van Den Ouwel FA, Valkenburg HA (1990). Do clinical findings associate with radiographic osteoarthritis of the knee. Ann Rheum Dis.

[3] Parker DA, Scholes C, Neri T (2018). Non-operative treatment options for knee osteoarthritis: current concepts. JISAKOS.

[4] Boesen M, Ellegaard K, Henriksen M, Gudbergsen H, Hansen P, Bliddal H (2017). Osteoarthritis year in review 2016: Imaging. Osteoarthritis Cartilage.

[5] Silva NC de OV e., Anjos RL dos, Santana MMC, Battistella LR, Alfieri FM. Discordance between radiographic findings, pain, and superficial temperature in knee osteoarthritis. Reumatologia. 2021;58(6):375–80. doi:10.5114/REUM.2020.102002 10.5114/reum.2020.102002 PMC7792543 33456080

[6] Cho HJ, Chang CB, Yoo JH, Kim SJ, Kim Kim TK (2010). Gender differences in the correlation between symptom and radiographic severity in patients with knee osteoarthritis. Clinical Orthopaedics and Related Research.

[7] Finan PH, Buenaver LF, Bounds SC, Hussain S, Park RJ, Haque UJ (2013). Discordance between pain and radiographic severity in knee osteoarthritis: Findings from quantitative sensory testing of central sensitization. Arthritis Rheum.

[8] Alami S, Boutron I, Desjeux D, Hirschhorn M, Meric G, Rannou F, Poiraudeau S. Patients’ and practitioners’ views of knee osteoarthritis and its management: a qualitative interview study. PLoS ONE. 2011;6(5):e19634. 10.1371/journal.pone.0019634. 10.1371/journal.pone.0019634 PMC3088707 21573185

[9] Making the best use of a department of clinical radiology: Guidelines for doctors 4 ed. 1998. Bibliographic information available from INIS: http://inis.iaea.org/search/search.aspx?orig_q=RN:31046779. Available from British Library Document Supply Centre- DSC:99/17633.

[10] Bedson J, Croft PR (2008). The discordance between clinical and radiographic knee osteoarthritis: A systematic search and summary of the literature. BMC Musculoskelet Disord.

[11] Zhang W, Doherty M, Peat G, Bierma-Zeinstra MA, Arden NK, Bresnihan B (2010). EULAR evidence-based recommendations for the diagnosis of knee osteoarthritis. Ann Rheum Dis.

[12] Altman R, Asch E, Bloch D, Bole G, Borenstein D, Brandt K (1986). Development of criteria for the classification and reporting of osteoarthritis. classification of osteoarthritis of the knee. diagnostic and therapeutic criteria committee of the american rheumatism association. Arthritis Rheum.

[13] Sakellariou G, Conaghan PG, Zhang W, Bijlsma JWJ, Boyesen P, D’Agostino MA (2017). EULAR recommendations for the use of imaging in the clinical management of peripheral joint osteoarthritis. Ann Rheum Dis.

[14] Bedson J, Jordan K, Croft P (2003). How do GPs use x rays to manage chronic knee pain in the elderly? A case study. Ann Rheum Dis.

[15] Ouellet D, Moffet H (2002). Locomotor deficits before and two months after knee arthroplasty. Arthritis Rheum.

[16] Zeni J, Higginson J (2009). Dynamic knee joint stiffness in subjects with a progressive increase in severity of knee osteoarthritis. Clin Biomech (Bristol, Avon).

[17] Zeni JA, Higginson JS (2009). Differences in gait parameters between healthy subjects and persons with moderate and severe knee osteoarthritis: A result of altered walking speed?. Clin Biomech.

[18] Chang A, Hayes K, Dunlop D, Hurwitz D, Song J, Cahue S (2004). Thrust during ambulation and the progression of knee osteoarthritis. Arthritis Rheum.

[19] Weidow J, Tranberg R, Saari T, Karrholm J (2006). Hip and knee joint rotations differ between patients with medial and lateral knee osteoarthritis: gait analysis of 30 patients and 15 controls. J Orthop Res.

[20] Omori G, Narumi K, Nishino K, Nawata A, Watanabe H, Tanaka M (2016). Association of mechanical factors with medial knee osteoarthritis: A cross-sectional study from matsudai knee osteoarthritis survey. J Orthop Sci.

[21] D’Souza N, Charlton J, Grayson J, Kobayashi S, Hutchison L, Hunt M, Simic M. Are biomechanics during gait associated with the structural disease onset and progression of lower limb osteoarthritis? A systematic review and meta-analysis. Osteoarthritis Cartilage. 2022;30(3):381–94. 10.1016/j.joca.2021.10.010. 10.1016/j.joca.2021.10.010 34757028

[22] Thorp LE, Sumner DR, Block JA, Moisio KC, Shott S, Wimmer MA (2006). Knee joint loading differs in individuals with mild compared with moderate medial knee osteoarthritis. Arthritis Rheum.

[23] Zeni J, Higginson J (2009). Differences in gait parameters between healthy subjects and persons with moderate and severe knee osteoarthritis: a result of altered walking speed?. Clin Biomech (Bristol, Avon).

[24] Wink AE, Gross KD, Brown CA, Guermazi A, Roemer F, Niu J (2017). Varus thrust during walking and the risk of incident and worsening medial tibiofemoral MRI lesions: the Multicenter Osteoarthritis Study. Osteoarthr Cartil.

[25] Miyazaki T, Wada M, Kawahara H, Sato M, Baba H, Shimada S (2002). Dynamic load at baseline can predict radiographic disease progression in medial compartment knee osteoarthritis. Ann Rheum Dis.

[26] Zeni JA, Flowers P, Bade M, Cheuy V, Stevens-Lapsley J, Snyder-Mackler L (2019). Stiff knee gait may increase risk of second total knee arthroplasty. J Orthop Res.

[27] Sharma L, Chang AH, Jackson RD, Nevitt M, Moisio KC, Hochberg M (2017). Varus Thrust and Incident and Progressive Knee Osteoarthritis. Arthritis Rheumatol.

[28] Therrien  M,  Fuentes  A, Landry  P, ElHachem  C, Pontbriand  R (2016). Real-world clinical result from a multimodal management program for knee osteoarthritis. Osteoarthr Cartil.

[29] Haim A, Rubin G, Rozen N, Goryachev Y, Wolf A, Salai M (2014). Six-week gait retraining program reduces knee adduction moment, reduces pain, and improves function for individuals with medial compartment knee osteoarthritis. J Orthop Res.

[30] Bowd J, Biggs P, Holt C, Whatling G (2019). Does gait retraining have the potential to reduce medial compartmental loading in individuals with knee osteoarthritis while not adversely affecting the other lower limb joints? A systematic review. Arch Rehabil Res Clin Transl.

[31] Cagnin A, Choinière M, Bureau NJ, Durand M, Mezghani N, Gaudreault N (2020). A multi-arm cluster randomized clinical trial of the use of knee kinesiography in the management of osteoarthritis patients in a primary care setting. Postgrad Med.

[32] Cagnin A (2020). Impact of a personalized care approach on 3D gait impairments in knee osteoarthritis patients (a cluster randomized controlled trial). Osteoarthritis Cartilage.

[33] Cagnin A (2019). Impact of a personalized home exercise program for knee osteoarthritis patients on 3d kinematics: A cluster randomized controlled trial. Osteoarthritis Cartilage.

[34] Calmbach WL, Hutchens M. Evaluation of patients presenting with knee pain: Part I. History, physical examination, radiographs, and laboratory tests. Am Fam Physician. 2003;68(5):907–12. 13678139

[35] Hunt MA, Birmingham TB, Jenkyn TR, Giffin JR, Jones IC (2008). Measures of frontal plane lower limb alignment obtained from static radiographs and dynamic gait analysis. Gait Posture.

[36] Clément J, Blakeney W, Hagemeister N, Desmeules F, Mezghani N, Lowry V (2019). Hip-Knee-Ankle (HKA) angle modification during gait in healthy subjects. Gait Posture.

[37] Brunnekreef JJ, van Uden CJ, van Moorsel S, Kooloos JG. Reliability of videotaped observational gait analysis in patients with orthopedic impairments. BMC Musculoskelet Disord. 2005;6:17. Published 2005 Mar 17. doi:10.1186/1471-2474-6-17. 10.1186/1471-2474-6-17 PMC555760 15774012

[38] Kellgren JH, Lawrence JS (1957). Radiological assessment of osteo-arthrosis. Ann Rheum Dis.

[39] Klara K, Collins JE, Gurary E, Elman SA, Stenquist DS, Losina E (2016). Reliability and accuracy of cross-sectional radiographic assessment of severe knee osteoarthritis: Role of training and experience. J Rheumatol.

[40] Damen J, Schiphof D, Wolde ST, Cats HA, Bierma-Zeinstra SMA, Oei EHG (2014). Inter-observer reliability for radiographic assessment of early osteoarthritis features: The CHECK (cohort hip and cohort knee) study. Osteoarthr Cartil.

[41] Lustig S, Magnussen RA, Cheze L, Neyret P (2012). The KneeKG system: A review of the literature. Knee Surg, Sport Traumatol Arthrosc.

[42] Roos EM, Roos HP, Lohmander LS, Ekdahl C, Beynnon BD (1998). Knee Injury and Osteoarthritis Outcome Score (KOOS)–development of a self-administered outcome measure. J Orthop Sports Phys Ther.

[43] Roos EM, Toksvig-Larsen S (2003). Knee injury and Osteoarthritis Outcome Score (KOOS) - validation and comparison to the WOMAC in total knee replacement. Health Qual Life Outcomes.

[44] Ornetti P, Parratte S, Gossec L, Tavernier C, Argenson JN, Roos EM, Guillemin F, Maillefert JF (2008). Cross-cultural adaptation and validation of the French version of the Knee injury and Osteoarthritis Outcome Score (KOOS) in knee osteoarthritis patients. Osteoarthritis Cartilage.

[45] Hotelling H (1936). Relations Between Two Sets of Variates. Biometrika.

[46] Sherry A, Henson RK (2005). Conducting and interpreting canonical correlation analysis in personality research: a user-friendly primer. J Pers Assess.

[47] Rosentha l R, Rubin D, Meng XL. Comparing Correlated Correlation Coefficients. Psychological Bulletin. 1992;111(No. 1):172–5.

[48] Steiger JH (1980). Tests for comparing elements of a correlation matrix. Psychol Bull.

[49] R Core Team (2021). R: A language and environment for statistical computing. R Foundation for Statistical Computing, Vienna, Austria. URL https://www.R-project.org/.

[50] Boonstra MC, Schwering PJ a, Malefijt MC De Waal, Verdonschot N (2010). Sit-to-stand movement as a performance-based measure for patients with total knee arthroplasty. Phys Ther.

[51] Bensalma F, Mezghani N, Ouakrim Y, Fuentes A, Choinière M, Bureau NJ (2019). A multivariate relationship between the kinematic and clinical parameters of knee osteoarthritis population. Biomed Eng Online..

[52] Naili JE, Esbjörnsson AC, Iversen MD (2017). The impact of symptomatic knee osteoarthritis on overall gait pattern deviations and its association with performance-based measures and patient-reported outcomes. Knee.

[53] Schiphof D, Kerkhof HJ, Damen J, de Klerk BM, Hofman A, Koes BW (2013). Factors for pain in patients with different grades of knee osteoarthritis. Arthritis Care Res (Hoboken).

[54] Son KM, Hong JI, Kim DH, Jang DG, Crema MD, Kim HA (2020). Absence of pain in subjects with advanced radiographic knee osteoarthritis. BMC Musculoskelet Disord.

[55] Marriott KA, Birmingham TB, Leitch KM, Pinto R, Giffin JR (2019). Strong independent associations between gait biomechanics and pain in patients with knee osteoarthritis. J Biomech.

[56] Fukui N, Yamane S, Ishida S, Tanaka K, Masuda R, Tanaka N (2010). Relationship between radiographic changes and symptoms or physical examination findings in subjects with symptomatic medial knee osteoarthritis: a three-year prospective study. BMC Musculoskelet Disord.

[57] Bensalma F, Richardson G, Ouakrim Y, Fuentes A, Dunbar M, Hagemeister N (2020). A Combined visualization method for multivariate data analysis application to knee kinematic and clinical parameters relationships. Appl Sci.

[58] Zeni JA, Flowers P, Bade M, Cheuy V, Stevens-Lapsley J, Snyder-Mackler L (2019). Stiff knee gait may increase risk of second total knee arthroplasty. J Orthop Res.

[59] Sharma L, Song J, Felson DT, Cahue S, Shamiyeh E, Dunlop DD (2001). The role of knee alignment in disease progression and functional decline in knee osteoarthritis. JAMA.

[60] Lane NE, Brandt K, Hawker G (2011). OARSI-FDA initiative: defining the disease state of osteoarthritis. Osteoarthritis Cartilage.

[61] Milan Meloun, Jiří Militký. 4 - Statistical analysis of multivariate data. Editor(s): Milan Meloun, Jiří Militký, Statistical Data Analysis, Woodhead Publishing India. 2011:151–403. ISBN 9780857091093. 10.1533/9780857097200.151.

